# Extra Virgin Olive Oil (EVOO): Quality, Safety, Authenticity, and Adulteration

**DOI:** 10.3390/foods10050995

**Published:** 2021-05-02

**Authors:** Theodoros Varzakas

**Affiliations:** Department Food Science and Technology, University of the Peloponnese, 24100 Kalamata, Greece; t.varzakas@uop.gr

The prevention and bioactivity effects associated with the so-called “Mediterranean diet” make olive oil the most consumed edible fat in the food intake of the Mediterranean basin.

The road to quality demands that legislation should be followed. Hence, official European Union classifications such as protected designation of origin (PDO), protected geographical indication (PGI), etc. guarantee the quality and the origin of the labeled foodstuff.

The profiling of volatile components and the aroma of olive oil are key factors in the quality dimension and are affected by various factors and conditions such as cultivar; atmospheric, pedologic, and fostering conditions; the ripening degree; olive and oil storage; and the technology of oil extraction from drupes, as well as the quality of the pre-extraction procedures.

In extra virgin olive oil production, as in all kinds of production, the maintenance of high quality standards is assured by the olive fruits’ and the final products’ quality. Modern milling technologies can aid in the direction of quality and safety, and thus can be employed in the production of extra virgin olive oil (EVOO), which can be directly consumed without any further manipulation. The overall quality of EVOO should be determined by quality characteristics including sensory analysis, stability, and nutritional value and safety (microbiology, absence of contaminants and toxins), along with authenticity.

Food authenticity issues are very important for the food industry due to legislation aspects, economics, quality specifications and conformance, safety concerns, and religious matters. Authentic EVOO should comply with the producer’s declaration regarding the quality of olive fruits, natural components, the absence of extraneous substances, production technology, the geographical and botanical origin, the production year, and the genetic identity. Hence, olive oil authenticity can be implemented by the validation of the application of accurate specifications for olive fruits and the selection of trustworthy suppliers with a quality assurance system in place. Adulteration is usually carried out for economical purposes such as the increase of the bulk volume, the increase in the yield of a process, or the advancing of the quality of a product of inferior quality by mixing. Authenticity methodologies will avoid adulteration but will also aid the control of accidental contaminations, e.g., in factories, where several oils are produced or used at the same time, or cross-contaminations.

In this Special Issue, both issues of olive fruits and EVOO have been investigated.

Virgin olive oil production parameters, composition, and quality were determined after storage for seven days at room temperature (RT), refrigerated, and frozen storage prior to oil production derived from post-harvest olive fruits (Istarska bjelica (IB) and Rosinjola (RO)). It was found that lower temperatures delayed the post-harvest maturation of IB fruits. Storage at RT maintained the highest oil yield and extractability index. Storage at RT increased the content of waxes, while the lower temperatures partially suppressed this phenomenon. Refrigerated storage preserved the concentration of the most phenolic compounds. Refrigeration seems to be the most suitable option for prolonged fruit storage [1].

Another study helped to prevent EVOO from fraud and adulteration and evaluated olive oil geographical origin by an open source visible and near infra-red (VIS-NIR) spectrophotometer [2]. They analyzed 67 Italian and 25 foreign EVOO samples, and multivariate analysis of variance (MANOVA) results reported significant differences (*p* < 0.001) between the Italian and foreign EVOO VIS-NIR matrices. They also employed an artificial neural network (ANN) model with an external test.

The next paper deals with the adulteration and authentication of extra virgin olive oil (EVOO) using vibrational spectroscopy signatures combined with pattern recognition analysis [3]. Oils were characterized by quality parameters such as fatty acid profile, free fatty acids (FFA), peroxide value (PV), pyropheophytins (PPP), and total polar compounds (TPC). Both techniques identified EVOO adulteration with vegetable oils, but Raman spectroscopy showed limited resolution detecting VOO/OO tampering. Excellent correlation was shown by partial least squares regression models.

The next work (H2020 OLEUM project) [4] represents the first published attempt to verify some of the recommended quality control tools for increasing harmonization among sensory panels. A new “decision tree” scheme was developed, and some IOC quality control procedures were applied. The adoption of these tools allowed for the reliable classification of 289 out of 334 VOOs. A “formative reassessment” was necessary to control misalignments. The authors reported the need to adopt new stable and reproducible reference materials in order to improve the panel’s skills and performance. They believe that sensory data need to be combined along with classification and characterization and correlation with physical–chemical data.

The following two studies come from Greece. The first one deals with the comparison and discrimination of two major monocultivar extra virgin olive oils from the two dominant olive cultivars in the southern region of Peloponnese, cv. Koroneiki and cv. Mastoides [5]. The fatty acid and sterolic profiles are used as compositional/traceability markers. This study aimed to evaluate the differences on specific chemical characteristics of the oils because of their botanical origin. Substantial compositional differences in the fatty acid and sterolic profiles between Koroneiki and Mastoides cultivars were detected by analysis of variance and principal component analysis.

The second study evaluated the extent to which Messinian olive oils comply with the “Kalamata Protected Designation of Origin (PDO)” regulation [6]. Quality indices were measured, and detailed analyses of sterols, triterpenic dialcohols, fatty acid composition, and wax content were conducted in a total of 71 samples of Messinian olive oils. Results demonstrated major fluctuations from the established EU regulatory limits on their chemical parameters. Results showed low concentrations of total sterols, high concentrations of campesterol, and a slight tendency towards high total erythrodiol content. Fatty acid composition and wax content were within the normal range.

The inter-varietal diversity of typical volatile and phenolic profiles of Croatian EVOO was investigated [7] to strengthen the varietal identities and position on the market of monovarietal and protected designation of origin (PDO) EVOO. 93 samples from six olive (*Olea europaea* L.) varieties were subjected to gas chromatography ion trap mass spectrometry (GC-IT-MS) and ultra-performance liquid chromatography with diode array detection (UPLC-DAD). Quantitative descriptive sensory analysis was also performed.

The last paper in this Special Issue deals with the quality of olive oil produced by different extractive processes carried out in a Tuscany oil mill at different harvesting periods in the same crop season, as affected by the presence of yeasts [8]. Yeast concentrations were higher in extraction processes at the end of the harvesting. Molecular methods were employed to identify twelve yeast species. Significant differences were shown by HS-SPME-GC-MS analysis of the volatile compounds in commercial EVOO inoculated with three yeast species (*Nakazawaea* *molendini-olei*, *Nakazawaea wickerhamii*, *Yamadazyma terventina*), and this was dependent on the strain inoculated. They also reported that the extraction plant might be colonized by some yeast species and this might affect the chemical and sensory characteristics of the EVOO.

The results of this Special Issue demonstrate the necessity for more stringent controls to increase quality and avoid fraud and adulteration of EVOO. In this direction, authentication methods are employed. It is quite important to safeguard quality and safety with various and promising tools that labs are equipped with, and sensory methodologies should be more critically combined with physico-chemical methods in order not only to comply with existing regulations, but also to provide consumers with a final product with added value. Companies should stop sacrificing value in the name of cost. Transparency procedures are essential in this direction.
